# Wnt/β-catenin signaling as a useful therapeutic target in hepatoblastoma

**DOI:** 10.1042/BSR20192466

**Published:** 2019-09-24

**Authors:** Ying-Li Sha, Shuang Liu, Wen-Wen Yan, Bo Dong

**Affiliations:** 1 Department of Pediatric, The First Hospital of Jilin University-The Eastern Division, Changchun, Jilin, China; 2 Department of Nursing Management, The First Hospital of Jilin University-The Eastern Division, Changchun, Jilin, China

**Keywords:** β-catenin, hepatoblastoma, NF-kB, TNF-α, Wnt

## Abstract

Hepatoblastoma is a malignant tumor in the liver of children that generally occurs at the age of 2–3 years. There have been ample evidence from the preclinical as well as clinical studies suggesting the activation of Wnt/β-catenin signaling in hepatoblastoma, which is mainly attributed to the somatic mutations in the exon 3 of β-catenin gene. There is increased translocation of β-catenin protein from the cell surface to cytoplasm and nucleus and intracellular accumulation is directly linked to the severity of the cancer. Accordingly, the alterations in β-catenin and its target genes may be used as markers in the diagnosis and prognosis of pediatric live tumors. Furthermore, scientists have reported the therapeutic usefulness of inhibition of Wnt/β-catenin signaling in hepatoblastoma and this inhibition of signaling has been done using different methods including short interfering RNA (siRNA), miRNA and pharmacological agents. Wnt/β-catenin works in association with other signaling pathways to induce the development of hepatoblastoma including Yes-associated protein (YAP)1 (YAP-1), mammalian target of rapamycin (mTOR) 1 (mTOR-1), SLC38A1, glypican 3 (GPC3), nuclear factor κ-light-chain-enhancer of activated B cells (NF-kB), epidermal growth factor receptor, ERK1/2, tumor necrosis factor-α (TNF-α), regenerating islet-derived 1 and 3 α (REG1A and 3A), substance P (SP)/neurokinin-1 receptor and PARP-1. The present review describes the key role of Wnt/β-catenin signaling in the development of hepatoblastoma. Moreover, the role of other signaling pathways in hepatoblastoma in association with Wnt/β-catenin has also been described.

## Introduction

Hepatoblastoma is a malignant tumor in the liver of children that generally occurs at the age of 2–3 years [1]. Hepatoblastoma is of different subtypes, differentiated on the basis of histological features and these include fetal, embryonal, macrotrabecular and small cell subtype [2]. Due to its poor clinical prognosis and availability of limited chemotherapeutic agents, there is a need of identifying new therapeutic targets for the effective management of hepatoblastoma. Genomic studies have identified the heterogeneity in genetic mutations in hepatoblastoma patients. In approximately 8% cases of hepatoblastoma, the missense, deletion or insertional mutations have been recorded in AXIN1, but not in AXIN2, genes [3]. Moreover, changes in the telomerase reverse transcriptase have also been reported in some of the hepatoblastoma patients, whose clinical prognosis is particularly unfavorable [4]. Nevertheless, the mutations in CTNNB1 genes (β-catenin) are most frequently observed in hepatoblastoma patients (Haines et al., 2019) and an increase in cytoplasmic and nuclear immunostaining of β-catenin has been documented in these cases of hepatoblastoma [5,6].

The Wnt/β-catenin signaling pathway is crucial in controlling hepatic homeostasis, maintaining adherens junctions, metabolic zonation and regeneration suggesting its role in almost every aspect related to liver functioning. However, its aberrant activation is associated with the development of various hepatic diseases including hepatoblastoma [7–13]. Indeed, it is reported that full-length β-catenin is the predominant form responsible for early liver development. However, calpain-mediated cleavage of β-catenin yields transcriptionally active 75-kDa truncated β-catenin (lacking 95 N-terminal amino acids), which is translocated to the cytoplasm and nucleus to induce the development of hepatoblastoma [14]. The activation of β-catenin in fetal liver progenitor cell is sufficient to induce hepatoblastoma [15].

Since β-catenin expression has been detected in all the heterologous elements such as squamous, osteoid and chrondroid tissues, along with the mesenchymal derived cells, therefore, scientists have employed the β-catenin staining to differentiate different histological variants of hepatoblastoma [16–18]. Studies have also shown that the nuclear β-catenin localization is directly related to poor differentiation and its localization is significantly higher in the embryonal and undifferentiated type of hepatoblastoma than fetal type of hepatoblastoma [19,20]. Moreover, an increase in nuclear β-catenin is also positively correlated with increased positive staining of cyclin D1, a nuclear factor protein controlling cellular proliferation [21]. The present review describes the key role of Wnt/β-catenin signaling in the development of hepatoblastoma. Moreover, the role of other signaling pathways in hepatoblastoma in association with Wnt/β-catenin has also been described.

## Mutations in β-catenin gene leads to its intracellular accumulation in the patients of hepatoblastoma

The studies in late 1990s reported that the mutations in the β-catenin genes lead to accumulation of β-catenin protein in the cytoplasm and nucleus of the cells of hepatoblastoma. Bläker et al. [22] reported an increase in the β-catenin levels in the cytoplasm and nuclei of three hepatoblastoma patients. Moreover, the authors also reported the presence of mutation in the exon 3 of the CTNNB1 (β-catenin gene) suggesting that the mutation in β-catenin gene may contribute in increasing its accumulation in tumor cells [22]. Based on the examination studies of 52 biopsies of hepatoblastoma, Koch et al. [23] reported that there are mutations in exon 3 encoding the degradation targeting box of β-catenin. Since the main role of degradation targeting box is to induce intracellular degradation of phosphorylated form of β-catenin, the mutations of these degradation targeting box may be responsible for increased accumulation of β-catenin protein in the cytoplasm and nucleus [23].

Later, scientists verified these initial findings and reported that somatic mutations in β-catenin gene are responsible for its accumulation inside the tumor cells, a crucial event in tumorigenesis [20,24–27]. Indeed, it is proposed that hepatoblastoma presents with the highest rate (50–90%) of β-catenin mutations [4,28]. In most of these cases, the mutations are either point mutations or interstitial deletions [3,29]. The sequence analysis of NH_2_-terminal domain of β-catenin gene in epithelial and mixed hepatoblastoma patients revealed the presence of missense mutations or interstitial deletions in the GSK-3β phosphorylation motif (Wei et al., 2000). The study performed by Takayasu et al. [21] in 68 primary hepatoblastoma patients revealed the missense mutations or deletions of β-catenin genes in approximately 44 (65%) tumors. It was also revealed that all mutations were somatic and were localized to the exon 3 of β-catenin gene, which encodes the amino acid residues involved in its degradation [21].

## Mutations in β-catenin gene and intranuclear accumulation of β-catenin in animal models of hepatoblastoma

Apart from the clinical studies, scientists have explored the role of mutations of β-catenin in different animal models of hepatoblastoma. In 19-anthraquinone-induced and 8-oxazepam-induced hepatoblastoma in B6C3F1 mice, mutations have been reported in the β-catenin gene. Moreover, the nuclear localization of β-catenin protein was identified in hepatoblastoma in comparison with normal liver cells, in which β-catenin was detected mainly on the plasma membrane [30]. In another study, the same group of scientists identified the increased expression of β-catenin in the nucleus in chemical-induced hepatoblastoma in B6C3F1 mice. Furthermore in chemical-induced hepatoblastoma model, an increase in intranuclear localization of β-catenin was correlated with increase in the expression of cyclin D1 and c-Jun, which are target genes of β-catenin and crucial in cellular proliferation. Moreover, there was a corresponding decrease in the membrane expression of E-cadherin, whose normal function is to interact with the β-catenin protein and retain it on the plasma membrane of the cell [31]. Another study has shown that hepatocytes with β-catenin nuclear translocation exhibit an increase in the expression of oncogenes (c-myc). Moreover, these hepatocytes exhibit abnormal cellular proliferation, metastatic behavior and auto-renewal capability suggesting that the activation of Wnt/β-catenin pathway is involved in tumor development [32].

## Increase in expression of proteins/components related to Wnt/β-catenin signaling

As described in above studies, scientists have directly measured the expression of β-catenin in the cytoplasm or nucleus in tumor cells to delineate its role in hepatoblastoma. Apart from it, the expression of other related proteins has also been used as an indirect method to identify the activation of Wnt signaling. It is well reported that four and a half of LIM-only protein 2 (FHL2) is a novel β-catenin-interacting protein and it acts as a co-activator of β-catenin. In hepatoblastoma patients, an increase in the expression of FHL2 has been detected, which suggests the activation of Wnt pathway. An increase in the expression of FHL2 along with β-catenin mutation suggests that FHL2 may be possibly involved in transactivating the β-catenin signaling in cancer cells [33]. Studies have shown the increase in the expression of endogenous Wnt antagonists as a common event in hepatoblastoma. Human Dickkopf-1 (hDkk-1) gene secretes protein, which acts as a potent inhibitor of the Wnt signaling pathway. A research study reported the increase in the expression of hDkk-1 in the biopsy specimens of hepatoblastoma patients [34]. Studies have shown the elevated expression of other Wnt antagonists, including Nkd-1 and β-TrCP in hepatoblastomas [35]. The overexpression of the Wnt antagonists may be possibly attributed to uncontrolled and excessive activation of Wnt signaling. In other words, an increase in endogenous Wnt antagonist in hepatoblastoma may be a compensatory mechanism to counter the excessive and uncontrolled activation of Wnt signaling [34].

## Association of Wnt/β-catenin with other signaling pathways

**Figure 1:**
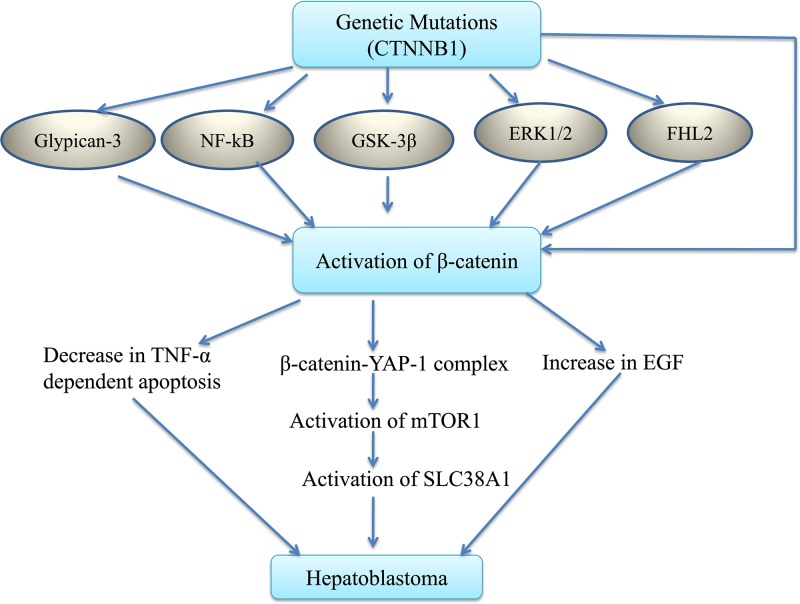
Different type of mutations including CTNNB1 gene may directly or indirectly activate the β-catenin signaling There may be activation of glypican-3, NF-kB, GSK-3β, ERK1/2 and/or co-activator of β-catenin, i.e. FHL2. Activation of β-catenin signaling may induce different pathways to induce the development of hepatoblastoma including inhibition of TNF-α-dependent apoptosis, increase in the secretion of epithelial growth factor (EGF) and formation of complex of β-catenin with YAP-1 protein. This interaction may activate mTOR pathway to increase the expression of SLC38A1 (amino acid transporter) that may be critical in the development of hepatoblastoma. Abbreviations: mTOR, mammalian target of rapamycin; TNF-α, tumor necrosis factor-α.

It is shown that Wnt/β-catenin works in association with other signaling pathways to induce the development of hepatoblastoma (Figure 1).

### Yes-associated protein 1 and mammalian target of rapamycin-1

Increasing number of studies has shown the cooperation between β-catenin and Yes-associated protein 1 (YAP1) in inducing the development of hepatoblastoma. In a clinical study, the nuclear co-localization of β-catenin and YAP1 was identified selectively in approximately 80% of the cases of hepatoblastoma, but not in hepatocellular carcinoma and cholangiocarcinoma [36]. Another experimental study has shown the co-expression of mutated form of β-catenin and YAP1 lead to activation of YAP and Wnt signaling, which then contributes in the development of hepatoblastoma [37]. Indeed, intravenous injection of plasmids coding for ΔN90-β-catenin and S127A-Yap1 has been shown to induce the development of hepatoblastoma in mice (Yap1-β-catenin model) within 5 weeks. In this model, tumor growth is closely associated with an increase in the nuclear expression of YAP1 and β-catenin [38]. In hepatoblastoma cell lines (HuH6, HepG2, HepT1, HC-AFW1, HepG2 and HC-AFW1) and in Yap1-β-catenin mice model, knockout of YAP-1 or β-catenin using short interfering RNAs (siRNAs) was shown to suppress cellular proliferation and tumorigenesis, respectively. Accordingly, it is proposed that β-catenin and YAP-1 interact physically to induce the development of hepatoblastoma [36]. A recent study has shown that YAP regulates downstream gene expression through interaction with the TEA domain (TEAD) proteins. The blockade of TEAD2 function was associated with a decrease in development of hepatoblastoma in mice and reduction in cellular proliferation in hepatoblastoma cell lines. On the other hand, activation of TEAD2 DNA-binding domain (by fusing with transcriptional activation domain) synergized with β-catenin and led to the development of hepatoblastoma in mice. Furthermore, the increased mRNA expression of TEAD4 in human hepatoblastoma tissues suggests that TEAD-mediated transcriptional activity may be crucial for YAP-β-catenin-induced development of hepatoblastoma [39].

In Yap1-β-catenin mice model, treatment with rapamycin (mammalian target of rapamycin (mTOR) inhibitor) was shown to attenuate the tumor growth over a period of time along with inhibition of mTORC1 suggesting that mTCOR1 is a potential target in YAP-1-β-catenin signaling cascade in hepatoblastoma [38]. A previous study also depicted the role of mTOR in the development of hepatoblastoma in association with YAP-1-β-catenin signaling cascade. Administration of mTOR1 inhibitor (MLN0128) and genetic ablation of *Raptor* (the unique subunit of mTORC1) was shown to inhibit cell growth in hepatoblastoma cells and delay YAP/β-catenin-induced development of hepatoblastoma in mice, respectively. Moreover at the molecular level, there was a parallel increase in the expression of amino acid transporter (SLC38A1) and mTORC1 in the hepatoblastoma tissues of mice, which was significantly reduced during mTOR1 inhibition. Silencing of YAP led to decrease in the SLC38A1 expression and inhibition of mTORC1 suggesting that the activation of YAP-β catenin may induce activation of mTORC1 to promote the development of hepatoblastoma by inducing the expression of SLC38A1 [40].

### Glypican 3

Glypican 3 (GPC3), a heparan sulfate proteoglycan, is a cell surface oncofetal protein, which is highly expressed in number of pediatric tumors including hepatoblastomas. Immunostaining has revealed the co-expression of GPC3 and β-catenin in the tissues of hepatoblastoma [41]. It is suggested that GPC3-induced activation of Wnt/β-catenin pathway is responsible for the development of hepatoblastoma [42]. Accordingly, scientists have developed antibodies against GPC3 and explored their usefulness in hepatoblastoma. Indeed, immunotoxin-based monoclonal antibody targeting GPC3 is reported to regress liver cancer in mice by inhibiting Wnt signaling [43]. Using a functional screening system, scientists reported the control of GPC3 expression in malignant liver cells through ten different miRNAs. Amongst these, the expression of miR-4510 was found to be inversely related to GPC3 expression and exogenous administration of this miR-4510 led to induction of apoptosis and blocked the tumor growth in an animal model. Apart from inhibition of GPC3 expression, miR-4510 also decreased the transcriptional activity of Wnt/β-catenin pathway again suggesting the interactions between GPC3 and Wnt/β-catenin in inducing hepatoblastoma [44].

### Nuclear factor κ-light-chain-enhancer of activated B cells

Nuclear factor κ-light-chain-enhancer of activated B cells (NF-kB) is an important gene, which plays a crucial role in the regulation of physiological and pathological processes, including cancer development [45]. To understand the interrelationship between Wnt signaling and NF-kB in hepatoblastoma, the effects of sequence-specific antisense morpholino oligonucleotides targeting pre-mRNA sequences of p50 and p65 subunits of NF-kB as well as of Wnt-1 genes were investigated. It was revealed that morpholino oligonucleotides inhibited NF-kB activation in human hepatoblastoma cell line (HepG2 cells) and decreased Wnt-1 production. Chromatin immunoprecipitation assay revealed the direct binding of NF-kB-p50 to the Wnt-1 promoter region. Accordingly, it may be suggested that the activation of NF-kB may increase the production of Wnt-1, which triggers the activation of Wnt/β-catenin to induce the development of hepatoblastoma [46].

### Epidermal growth factor receptor

The close relationship between epidermal growth factor receptors and β-catenin signaling has been explored in the development of hepatoblastoma. In transgenic mice with mutated β-catenin gene, an increase in hepatocyte proliferation along with increase in cytoplasmic and nuclear accumulation of β-catenin was demonstrated. Along with it, there was also a parallel increase in the mRNA and protein levels of epidermal growth factor receptors in the transgenic livers. The inhibition of epidermal growth factor receptors led to decrease in the liver size in these transgenic mice. More analysis confirmed the activation of epidermal growth factor receptor in response to Wnt-3A treatment, which was attenuated by, in the presence of Wnt antagonist, a frizzled related protein 1. Accordingly, it may be suggested that the activation of Wnt/β-catenin signaling increases the expression of epidermal growth factor receptors on the liver to increase proliferation of hepatocytes in hepatoblastoma [47].

### ERK1/2

Epithelial to mesenchymal transition (EMT) is a critical process in the progression of epithelial tumors including hepatoblastoma [48]. Mutations in the form of large deletions in exon 3 of β-catenin gene are shown to increase the nuclear accumulation of β-catenin and increase the expression of EMT factors including SNAIL, c-Met and vimentin showing the key role of β-catenin in EMT [49]. Furthermore, scientists have shown that there is a close relationship between ERK1/2 and β-catenin activation in inducing EMT in hepatoblastoma. Using 12-O-tetradecanoylphorbol-13-acetate (TPA), the signaling pathways involved in EMT of HepG2 cells was delineated. It was shown that TPA-induced phenotypic changes in the epithelial cells leading to spindle-shaped fibroblast-like morphology. There was also a loss of E-cadherin function, which in turn was shown to be dependent on ERK1/2 activation. Moreover, it was also shown that ERK1/2 activation is followed by a complex formation between Snail and β-catenin, which led to activation of Wnt pathway and loss of E-cadherin function to promote EMT in hepatocellular carcinoma cells [50].

### Others

There have been other molecules that participate in Wnt/β-catenin triggered signaling pathway in hepatoblastoma including tumor necrosis factor-α (TNF-α), regenerating islet-derived 1 and 3 α (REG1A and 3A), substance P (SP)/neurokinin-1 receptor and PARP-1. Using immortalized murine hepatocyte cell line (AML12) that stably expressed mutant β-catenin, it was shown that stabilization of β-catenin enhanced proliferation of hepatocytes, suppressed TNF-α-induced apoptosis, induced anchorage-independent cell growth and increased the expression of c-myc and cyclin D1. It suggests that increase in β-catenin may produce its cellular proliferative effects in hepatoblastoma due to inhibition of TNF-α-induced apoptosis [51]. REG1A and 3A proteins are involved in liver regeneration and proliferation. It has been shown that there is a close relation between REG1A/3A proteins and β-catenin in development of hepatoblastoma. Indeed, a correlation has been found between up-regulation of REG1A/3A and β-catenin status in hepatoblastomas and scientists delineated that REG1A/3A are the downstream targets of the Wnt pathway during liver tumorigenesis [52]. It has been shown that there is a close relationship between Wnt and SP/NK-1 receptor in the development of hepatoblastoma. Administration of SP/neurokinin-1 receptor agonist aprepitant in three human hepatoblastoma cell lines, i.e. HepT1, HepG2 and HuH6 was associated with decrease in cellular proliferation along with inhibition of Wnt pathway suggesting that activation of Wnt pathway is regulated by SP/neurokinin-1 signaling pathway [53]. Studies have shown that activation of PARP-1 may lead to post-translational modifications of tumor suppressor genes along with activation of WNT/β-catenin signaling to induce the development of hepatoblastoma. Moreover, inhibition of PARP1 was shown to normalize the expression of tumor suppressor genes, reduce cell proliferation and inhibit Wnt/β-catenin signaling [54].

## Inhibition of Wnt/β-catenin signaling leads to suppression of hepatoblastoma

Considering Wnt/β-catenin as a potential therapeutic target, scientists have attempted to explore the usefulness of suppression of Wnt/β-catenin signaling in the preclinical studies related to hepatoblastoma. In hepatoblastoma cell lines (HuH-6 and HepG2) transfection of siRNAs or sequence-specific antisense morpholino oligonucleotides leads to degradation of overexpressed β-catenin in the nucleus and reduces the levels of c-myc. It suggests that β-catenin may be potential target of gene therapy to manage pediatric hepatic tumors, with mutations and overexpression of β-catenin gene [46,55]. Transfection of hepatoblastoma cell lines (Huh-6 and HepG2) with siRNA of frizzled receptors (receptor of Wnt ligands) is shown to inhibit Wnt signaling, decrease the expression of frizzled receptor genes, cyclin D1 and suppress the cellular proliferation. It suggests that WNt/frizzled receptors may serve as useful therapeutic target for hepatoblastoma [56]. Another study has shown that pharmacological inhibition of β-catenin in the presence of celecoxib and ICG001 reduced the cell viability and decreased the nuclear β-catenin expression in cultivated hepatoblastoma cells [57]. ICG-001 is a novel small-molecule inhibitor of Wnt signaling, which acts by disrupting β-catenin-CREB binding protein interactions with IC_50_ ranging from 4.87 to 32 μmol/l [58]. Administration of naturally occurring flavonoid epigallocatechin-3-gallate was shown to inhibit the growth of hepatoblastoma in a time- and dose-dependent manner through inhibition of Wnt signaling. Epigallocatechin-3-gallate-mediated inhibition of Wnt signaling was delineated by down-regulation of Wnt-responsive reporter gene activity and decreased the expression of Wnt target genes, including c-myc. Moreover, epigallocatechin-3-gallate also led to induction of tumor suppressor gene (*SFRP1*), which normally down-regulates Wnt signaling and is transcriptionally silenced in hepatoblastoma cells [59]. Co-administration of ICG001 has been shown to enhance the anti-tumor activity of sorafenib in an animal model of hepatoblastoma [60]. The scientists have developed miRNA mimetics capable of inhibiting *CTNNB1* expression. Among the different mimetics, miR-624-5p has been shown to trigger cell senescence in *in vitro* as well as *in vivo* studies involving experimental hepatoblastoma growth along with inactivation of Wnt/β-catenin signaling [61]. Therefore, it may be proposed that inhibition of Wnt/β-catenin signaling through siRNA, pharmacological modulators or miRNA may therapeutically exploited to effectively manage hepatoblastoma.

## Discussion

Based on the number of studies, it has been proposed that Wnt/β-catenin signaling pathway may be an important target to modulate the proliferation of cancer cells including cancers of female reproductive system [62] and breast cancer [63]. A correlation has been identified between the overexpression of β-catenin and poor prognosis of breast cancer patients [64]. In patients suffering from gastric cancers, a significant increase in the expression of YAP and β-catenin has been correlated to the disease severity [65]. An activation of Wnt/β-catenin signaling has also been associated with the development of colorectal cancer [66], glioblastoma [67] and bladder cancer [68] in patients. Considering the importance of this signaling in different cancers, a Phase II trial has been designed to investigate the safety and efficacy of oral niclosamide, Wnt/β-catenin signaling modulating drug, in colorectal cancer patients [69]. Moreover, it is suggested that the levels of endogenous negative regulators of Wnt/β-catenin including DKK1 and secreted frizzled-related protein-1 may be potentially employed as biomarkers for evaluating the beneficial effects of exercise on the metabolism and prognosis of breast cancer patients [70].

There has been an exploration of new targets in the management of hepatoblastoma including signal transducer and activator of transcription signaling 3 (STAT3) [71,72]; NOTCH receptors and their ligands [73]; PI3K/Akt, ERK and p38 signaling pathways [74]; GPC3 [75]; PIM Kinases [76]; ROCK1 [77]; mTOR and YAP [38]. Among these targets, Wnt/β-catenin has been more explored in different types of cancers including hepatoblastoma. Moreover, this signaling pathway interconnects other targets including ERK1/2, glypican, mTOR and YAP in the development and progression of hepatoblastoma. There are a number of studies showing that hyperactivation of Wnt/β-catenin pathway may be critical in the development of hepatoblastoma. Indeed, it is postulated that the increase in mutation of β-catenin gene on exon 3, i.e. CTNNB1 is responsible for increase in translocation of β-catenin from the plasma membrane to cytoplasm and nucleus [25,26,78]. An increase in activation of Wnt/β-catenin signaling may also be secondary to activation of GPC3, NF-kB, GSK-3β, ERK1/2 and co-activator of β-catenin, i.e. FHL2 [36,42,46]. The activation of β-catenin signaling may trigger the activation of other signaling pathways to induce the development of hepatoblastoma, including inhibition of TNF-α-dependent apoptosis, increase in the secretion of epithelial growth factor and formation of complex of with YAP-1 protein [40,47,51]. The interaction between β-catenin and YAP-1 protein may activate mTOR pathway to increase the expression of amino acid transporter, i.e. SLC38A1 [38], which may be critical in the development of hepatoblastoma.

Apart from the association of Wnt/β-catenin signaling with the induction of hepatoblastoma, studies have shown that the activation of this signaling also regulates hallmarks of tumor progression including degree of differentiation, invasiveness, metastasis [79]. The down-regulation of disheveled-2, a critical component of Wnt/β-catenin signaling pathway, has been associated with a decrease in the invasiveness of hepatoblastoma [80]. Using immunohistochemistry, a strong nucleocytoplasmic signal of β-catenin has been identified in the hepatoblastoma cells metastasized to lungs [81]. Moreover, the poorly differentiated subtypes of hepatoblastoma including embryonal and small cell undifferentiated (SCU) exhibit intense nuclear staining of β-catenin. On the other hand, the well-differentiated fetal (WDF) subtype of hepatoblastoma exhibit membranous staining of β-catenin [11,23,78]. It again suggests that activation of β-catenin signaling is associated with more aggressive tumor growth and poor prognosis. Accordingly, the inhibition of Wnt/β-catenin signaling using siRNA, miRNA or specific/non-specific blockers of Wnt and β-catenin may potentially attenuate the development and progression of hepatoblastoma.

## Conclusion

Somatic mutations in the exon 3 of β-catenin gene (CTNNB1) may lead to activation of Wnt/β-catenin signaling in hepatoblastoma, which is characterized by increased cytoplasmic/nuclear accumulation of β-catenin. The accumulation may result either directly or indirectly through other signaling pathways involving activation of GPC3, NF-kB, GSK-3β, ERK1/2 and FHL2, which serves as a co-activator of β-catenin. The activation of β-catenin signaling may trigger the activation of other signaling pathways to induce the development of hepatoblastoma, which may include inhibition of TNF-α-dependent apoptosis, increase in the secretion of epithelial growth factor and formation of complex of with YAP-1 protein. The interaction between β-catenin and YAP-1 protein may activate mTOR pathway to increase the expression of amino acid transporter, i.e. SLC38A1, which may be critical in the development of hepatoblastoma. The inhibition of Wnt/β-catenin signaling using siRNA, miRNA or pharmacological agents (specific or non-specific) may be potentially employed for the attenuating the progression of hepatoblastoma.

## Abbreviations

EMT: epithelial to mesenchymal transition
FHL2: four and a half of LIM-only protein 2
GPC3: glypican 3
hDkk-1: human Dickkopf-1
mTOR: mammalian target of rapamycin
NF-kB: nuclear factor κ-light-chain-enhancer of activated B cells
REG1A and 3A: regenerating islet-derived 1 and 3 α
siRNA: short interfering RNA
SP: substance P
TEAD: TEA domain
TNF-α: tumor necrosis factor-α
TPA: 12-O-tetradecanoylphorbol-13-acetate
YAP: Yes-associated protein
